# Impact of Fatty Acid Types and Microwave Post-Treatment on the Physicochemical Properties of Water Caltrop Starch–Lipid Complexes

**DOI:** 10.3390/foods14132254

**Published:** 2025-06-25

**Authors:** Pei-Chang Lee, Lih-Shiuh Lai

**Affiliations:** Department of Food Science and Biotechnology, National Chung Hsing University, 145 Xingda Rd., South Dist., Taichung City 402202, Taiwan; a0939394109@gmail.com

**Keywords:** starch–lipid complex, microwave post-treatment time, saturated fatty acid, unsaturated fatty acid

## Abstract

This study investigates the effects of microwave post-treatment and fatty acid type on the physicochemical properties of starch–lipid complexes derived from water caltrop (*Trapa taiwanensis* Nakai) starch. Complexes were prepared using stearic acid (C_18:0_) or oleic acid (C_18:1_), followed by microwave post-treatment at varying durations. Morphological analysis revealed that the starch–stearic acid complex exhibited more plate-like structures and birefringent spots compared to the starch–oleic acid complex. The complexing index increased with extended microwave exposure, indicating enhanced complex formation. Fourier-transform infrared spectroscopy showed no significant variation in the 1047/1022 cm^−1^ absorption ratio, suggesting that the short-range molecular order remained unaffected. However, X-ray diffraction analysis indicated increased relative crystallinity, particularly in the stearic acid complex (10.4%) compared to the oleic acid complex (4.8%), likely due to the higher linearity and saturation of stearic acid. Differential scanning calorimetry confirmed the presence of both type I and type II crystallization in all samples. The starch–stearic acid complex exhibited greater thermal stability, promoted type II crystallization, and enhanced the ordered structure of type I crystallization. In contrast, microwave treatment had limited influence on the thermal properties of the starch–oleic acid complex. These findings demonstrate that microwave post-treatment facilitates starch–lipid complex formation and improves structural organization, particularly when saturated fatty acids are employed.

## 1. Introduction

Water caltrop (*Trapa taiwanensis* Nakai) is an annual, herbaceous aquatic plant that primarily grows in wetlands of temperate climates. The kernel, which contains up to 80% starch on a dry basis, is the most commonly consumed part of the plant [1]. Among various starchy crops, water caltrop has gained attention for its unique characteristics, including a high gelatinization temperature and a high content of resistant starch [2].

Resistant starch resists digestion by enzymes in the human gastrointestinal tract, thereby helping to moderate postprandial blood glucose levels [3]. Consequently, it may offer potential benefits in managing obesity, type 2 diabetes, and cardiovascular disease [4]. The starch–lipid complex, formed by the interaction between amylose and guest molecules such as lipids, emulsifiers, or aromatic compounds, is classified as a novel type of resistant starch, designated resistant starch type 5 (RS5) [5], and is associated with the induction of a single-helix conformation in amylose [6]. Furthermore, microorganisms in the colon can ferment starch–lipid complexes to produce short-chain fatty acids (SCFAs), such as propionic and butyric acids, which play a critical role in modulating immune responses [7]. The benefits of starch–lipid complexes extend beyond improved digestibility and chronic disease management. Their higher thermal stability, compared to other forms of resistant starch, significantly enhances their suitability for food processing. Additionally, starch–lipid complexes can retard starch retrogradation and enhance the stability of frozen foods [8]. These characteristics position starch–lipid complexes as promising agents in the health and food industries.

Amylose consists primarily of linear chains of α-(1→4)-linked glucose units, whereas amylopectin is a highly branched polysaccharide containing both α-(1→4) and α-(1→6) glycosidic linkages. The relative abundance and structural configuration of these two components critically influence the formation and physicochemical properties of starch–lipid complexes. Numerous studies have demonstrated that starches with elevated amylose levels facilitate the formation of these complexes due to amylose’s propensity to adopt single-helical conformations that accommodate lipid inclusion [9,10,11]. Conversely, the branched architecture of amylopectin, characterized by short side chains, sterically hinders the formation of stable helical structures in the backbone, thereby impeding effective lipid binding [12]. Beyond starch type, the physicochemical characteristics of the lipid, particularly chain length and degree of saturation, along with processing parameters such as temperature, moisture content, and shear conditions, play a critical role in determining the structural and functional properties of starch–lipid complexes. Numerous studies have investigated the impact of lipid saturation on complex formation, though results have been inconsistent. Raza, et al. [13] reported that linoleic acid, due to its higher degree of unsaturation compared to stearic acid, forms amylose complexes with a higher complexing index, greater relative crystallinity, and increased resistant starch content. In contrast, Cervantes-Ramírez, et al. [14], using extrusion processing, found that only stearic acid interacted with corn starch to alter its crystalline structure, as evidenced by X-ray diffraction analysis.

Microwaves are electromagnetic waves with frequencies around 5 × 10^9^ Hz, falling between infrared and radio waves. These waves generate oscillating electric fields that cause polar molecules to vibrate, leading to frictional heating and a subsequent rise in temperature [15]. Microwaving is widely employed in food processing not only for its simplicity, rapid heating, and uniform energy distribution, but also as a physical method to modify starch structure, affecting properties such as granule morphology, crystallinity, swelling capacity, and solubility [16]. Furthermore, previous studies have reported that increasing microwave power can affect both α-(1,4)- and α-(1,6)-glycosidic linkages in amylopectin, resulting in the formation of relatively linear starch fragments or shorter glucan chains resembling amylose. This structural modification is often associated with a significant increase in the apparent amylose content [9]. However, the application of microwave technology to the formation and characterization of starch–lipid complexes remains relatively underexplored. Therefore, this study aims to investigate the effects of fatty acid saturation—comparing stearic acid (saturated) and oleic acid (unsaturated)—and microwave post-treatment duration on the structural and functional characteristics of water caltrop starch–lipid complexes.

## 2. Materials and Methods

### 2.1. Materials

Fresh water caltrop (*Trapa taiwanensis* Nakai), specifically the two-spiked variety, was procured from the local farmer in Xiaying District of Tainan City, Taiwan. Stearic acid (purity >98.5%) and oleic acid (purity 99%) were obtained from Sigma-Aldrich (St. Louis, MO, USA). All other chemicals used were of analytical grade.

### 2.2. Preparation of Water Caltrop Starch

Following the protocol established by Hung and Lai [17], approximately 250 g of water caltrop kernels were homogenized with 500 mL of ultrapure water (*w*/*w* ratio 1:2) using a laboratory-grade food processor (Blender LB10XS, Waring Laboratory Science, Torrington, CT, USA). The homogenization process comprised an initial low-speed blending phase at 300 rpm for 40 s, followed by a high-speed phase at 600 rpm for 20 s. This two-step blending cycle was repeated three times to ensure thorough disruption of the kernel matrix. The resulting slurry was transferred to a filtration cloth, and the starch-rich liquid fraction was extracted via mechanical pressing. The crude extract was subsequently passed through a 100-mesh stainless steel sieve to eliminate coarse particulates. The filtrate was allowed to stand at 4 °C to facilitate sedimentation of starch granules. Once the supernatant was decanted, the starch pellet was resuspended in a 0.1% (*w*/*v*) sodium hydroxide (NaOH) solution at a starch-to-solution volume ratio of 1:2 and incubated at 4 °C for 12 h to facilitate the removal of residual proteins and pigments. Following alkaline treatment, the starch was repeatedly washed with deionized and ultrapure water until the pH of the washing solution reached neutrality (pH 7.0). The purified starch was then collected, dried in a convection oven at 40 °C until the moisture content was reduced to below 10%, and milled using a laboratory grinder (RM100, Retsch, Haan, Germany) to a fine powder capable of passing through a 100-mesh sieve. The final starch product was stored in sealed containers at ambient temperature for subsequent analyses.

The chemical composition of the isolated starch was determined according to standard methods established by the Association of Official Analytical Chemists (AOAC). The crude protein, crude lipid, crude fiber, and ash contents were 0.36%, 0.14%, 0.00%, and 0.15%, respectively, indicating a high level of starch purity. Amylose content was determined using a commercial Amylose/Amylopectin Assay Kit (Cat. No. K-AMYL; Megazyme, Bray, Ireland), following the manufacturer’s instructions, and was found to be 22.03%.

### 2.3. Preparation of Water Caltrop Starch–Lipid Complex

A total of 15 g of water caltrop starch was dispersed in 300 mL of deionized water. The suspension was heated in a 90 °C water bath with continuous stirring for 30 min to ensure complete gelatinization of the starch. Subsequently, 1.5 g of fatty acids, pre-dissolved in 10 mL of 95% ethanol, was added to the gelatinized starch solution. The mixture was then transferred to a constant-temperature shaking water bath maintained at 90 °C and incubated with agitation for an additional 30 min to facilitate complex formation. The starch–fatty acid complexes were then subjected to microwave treatment using a domestic microwave oven (NN-ST34NB, Panasonic, Tokyo, Japan) operated at 700 W for varying durations: 0 min (control), 1 min, 2 min, and 3 min. After microwave treatment, the global temperature of the sample was measured using a digital thermometer (DE-3003, DEREE, New Taipei City, Taiwan). Then, 200 mL of 95% ethanol was added to the mixture and stirred thoroughly to extract unbound fatty acids. The resulting suspension was centrifuged at 10,000× *g* for 20 min, after which the supernatant was discarded and the precipitate was spread onto a tray and dried in a convection oven at 40 °C for 72 h. The dried material was ground, sieved through a 60-mesh screen, and stored in a desiccator until further use. The control gelatinized starch was prepared using the same procedure, except without the addition of the fatty acid ethanol solution.

### 2.4. Polarized Light Microscopy

A small quantity of starch was mixed with a drop of 50% glycerol solution on a microscope slide and covered with a cover slip at a 45° angle to minimize air bubble formation. The starch granules were observed under an optical microscope (BX41, Olympus Corporation, Tokyo, Japan) at 40× magnification. A polarizing microscope (BX-POL, Olympus Corporation, Tokyo, Japan), equipped with a camera adapter (U-CMAD 3, Olympus Corporation, Tokyo, Japan) and connected to a microphotography system (MC 4K, Ostec, Guangzhou, China), was used to capture images. Starch granule morphology was documented using an imaging software (KoPa Capture Version 8.5, Ostec Opto-electronic Co., LTD., Guangzhou, China). An objective micrometer (AX0001, Olympus Corporation, Tokyo, Japan) was used under the same magnification for calibration and measurement of granule size.

### 2.5. Scanning Electron Microscopy (SEM)

The sample was mounted on the platform using conductive double-sided adhesive tape. A platinum thin film was sputter-coated onto the sample surface using a metal ion coater (JEC-3000FC, JEOL, Tokyo, Japan) under a current of 10 mA for 30 s. The coated sample was then examined using a scanning electron microscope (JSM-7800F, JEOL, Tokyo, Japan) at an accelerating voltage of 3.0 kV and a magnification of 2000×.

### 2.6. Complex Index

Based on a previously described method with modifications [18], an iodine solution was prepared as a color reagent by dissolving 2 g of potassium iodide and 1.3 g of iodine in a small amount of warm water, then diluting the solution to a final volume of 100 mL with distilled water. A 200 mg sample was weighed into a 50 mL centrifuge tube, followed by the addition of 40 mL of distilled water. The mixture was homogenized using a vertical test tube mixer for 15 min. After mixing, the sample was centrifuged at 7200× *g* for 15 min. Then, 0.3 mL of the resulting supernatant was transferred to a new container, and 3 mL of iodine solution was added. The mixture was shaken for 1 min, and the absorbance was measured at 690 nm using a spectrophotometer (U-2800, Hitachi, Tokyo, Japan). The complex index was calculated using the following equation, with gelatinized starch serving as the reference.

$$Complex\;index\;(\%)=\frac{ABS\;of\;reference-ABS\;of\;sample}{ABS\;of\;reference}\times 100$$

### 2.7. X-Ray Diffraction (XRD) Analysis

A 2 g sample was weighed and placed in a glass desiccator maintained at 100% relative humidity for 24 h to equilibrate the sample structure prior to analysis. An X-ray diffractometer (X’Pert Pro MPD, Panalytical, Almelo, The Netherland) was used to determine the crystallinity of the sample. The X-ray diffraction (XRD) parameters were set as follows: voltage of 40 kV, current of 40 mA, scanning speed of 2°/min, and scanning range from 3° to 50° (2θ). Relative crystallinity was calculated using the following formula.

$$Relative\;crystallinity\;(\%)=\frac{Ic}{Ic+Ia}\times 100$$

where Ic and Ia indicate the integral diffraction intensity of the crystalline and the amorphous portion of the sample, respectively.

### 2.8. Fourier-Transform Infrared Spectroscopy (FTIR)

A small amount of starch powder was placed in an Eppendorf tube and dried in an oven at 40 °C for 24 h to remove free water. The sample was then analyzed using Fourier Transform Infrared Spectroscopy (FTIR, Bruker Vertex 70V, Hyperion 3000, 64 × 64 MCT Focal plane Array, Bruker Scientific LLC, Billerica, MA, USA) equipped with an attenuated total reflection (ATR) accessory. The measurement parameters were set to a wavenumber range of 4000–650 cm^−1^, 64 scans, and a spectral resolution of 4 cm^−1^.

### 2.9. Differential Scanning Calorimetry (DSC)

Differential scanning calorimetry (DSC) was employed to characterize the thermal properties of the samples. A dried sample weighing 3.2 mg was combined with 12.8 mg of deionized water, resulting in a total weight of 16 mg and a sample-to-water ratio of 1:4. The mixture was placed into a 40 μL aluminum pan (ME-277331, Mettler Toledo, Greifensee, Switzerland), hermetically sealed, and equilibrated at room temperature for 12 h to ensure moisture uniformity prior to analysis. DSC measurements were performed by heating the sample from 30 °C to 130 °C at a rate of 10 °C/min. An empty sealed aluminum pan was used as the reference under identical conditions. The onset temperature (T_o_), peak temperature (T_p_), conclusion temperature (T_c_), and enthalpy change (ΔH) of the phase transition were recorded.

### 2.10. Statistical Analysis

Variance analysis was performed using SPSS (version 19, IBM Corp., Armonk, NY, USA). Differences between means were evaluated using Duncan’s multiple range test, with *p* < 0.05 considered statistically significant.

## 3. Results and Discussion

### 3.1. Polarized Light Microscopic Features

**Figure 1:**
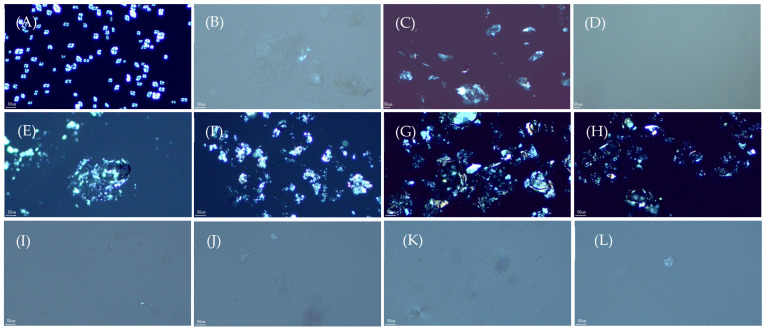
Polarized light microscopy images of native water caltrop starch, gelatinized starch, fatty acids, and microwave post-treated water caltrop starch–lipid complexes (scale bar = 50 μm). (**A**) Native water caltrop starch, (**B**) gelatinized water caltrop starch, (**C**) stearic acid (**D**) oleic acid (**E**) MS 0, (**F**) MS 1, (**G**) MS 2, (**H**) MS 3, (**I**) MO 0, (**J**) MO 1, (**K**) MO 2, and (**L**) MO 3, where “MS” denotes water caltrop starch–stearic acid complex, and “MO” denotes water caltrop starch–oleic acid complex, respectively. The number after MS and MO denotes the duration of microwave post-treatment in minutes.

Figure 1 displays the observations of native and gelatinized water caltrop starch, fatty acids, and various starch–lipid complexes under a polarized light microscope. A typical Maltese cross pattern was observed in native water caltrop starch, indicative of the birefringence associated with crystalline regions formed by the ordered arrangement of amylopectin side chains within the starch granules. When starch granules are heated in the presence of sufficient water and thermal energy, water penetrates the granular structure and, together with heat, disrupts the crystalline regions of the starch. This disruption results in the loss of birefringence and the disappearance of the Maltese cross pattern. However, slight bright spots were observed in gelatinized water caltrop starch under a polarizing microscope, which may be attributed to complexes formed between amylose and ethanol during the ethanol washing step following gelatinization. Additionally, the weak crystallinity observed in gelatinized starch may also result from retrogradation upon cooling to room temperature, followed by subsequent drying. The addition of stearic acid and oleic acid resulted in varying birefringence intensities among the samples under polarized light, with the starch–stearic acid complex exhibiting significantly higher birefringence than the starch–oleic acid complex. Nevertheless, the consistent presence of birefringence in all samples suggests that hydrophobic interactions between amylose and fatty acids promoted the formation of self-assembled starch–lipid complexes with V-type crystalline structures [18]. The greater number of bright spots observed in the stearic acid group suggests a higher extent of complex formation between water caltrop starch and stearic acid. These bright spots may also, in part, originate from uncomplexed (free) stearic acid, which, as demonstrated by Zaliha, et al. [19] and confirmed in the present study, exhibits birefringence under polarized light when in the solid state below its melting point.

### 3.2. Scanning Electron Microscopic (SEM) Features

**Figure 2:**
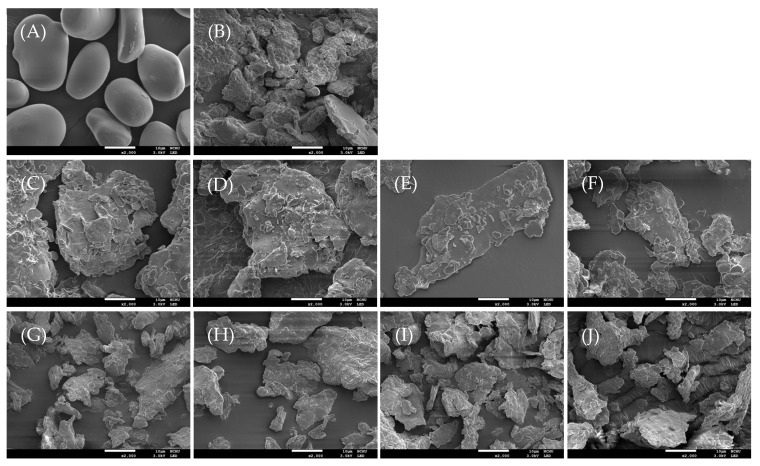
Scanning electron microscopy images of native water caltrop starch, gelatinized starch, and microwave post-treated water caltrop starch–lipid complexes (scale bar = 10 μm). (**A**) Native water caltrop starch, (**B**) gelatinized water caltrop starch, (**C**) MS 0, (**D**) MS 1, (**E**) MS 2, (**F**) MS 3, (**G**) MO 0, (**H**) MO 1, (**I**) MO 2, and (**J**) MO 3, where “MS” denotes water caltrop starch–stearic acid complex, and “MO” denotes water caltrop starch–oleic acid complex, respectively. The number after MS and MO denotes the duration of microwave post-treatment in minutes.

Figure 2 presents the scanning electron micrographs of native and gelatinized water caltrop starch, as well as various starch–lipid complexes. Native water caltrop starch granules appear oval-shaped with smooth surfaces. Upon heating at 90 °C for 30 min, the semi-crystalline structure within the granules is disrupted, ultimately leading to granule rupture. In samples containing stearic acid or oleic acid, irregular and flaky surface morphologies are observed, qualitatively indicating successful complexation of both fatty acids with water caltrop starch [20,21]. Moreover, the water caltrop starch–stearic acid complex samples (MS group) generally exhibit a greater number of parallel flaky structures on the starch surface, which may be associated with the quantity and size of the complexes formed. Kang et al. [21] reported that the abundance of parallel flaky structures is positively correlated with the amount of complex formed. In addition, in a study of the complexes formed between corn starch and stearic acid and oleic acid, Wu, et al. [22] also found that the complexes formed with oleic acid had a smaller particle size than those formed with stearic acid, suggesting that their presence is more difficult to be observe under scanning electron microscopy.

### 3.3. Complexation Effectiveness by Complex Index Evaluation

The ability of amylose to bind with iodine is significantly reduced after forming complexes with fatty acids via hydrophobic interactions [23]. Based on this characteristic, the difference in absorbance values following iodine staining between gelatinized starch and the starch–lipid complex can be used to quantify the extent of complex formation. As shown in Table 1, the water caltrop starch–stearic acid complex without microwave post-treatment (MS 0 group) exhibits a higher complex index value compared to the water caltrop starch–oleic acid complex without microwave post-treatment (MO 0 group). This suggests that stearic acid, a saturated fatty acid, has a greater propensity to form complexes with water caltrop starch than oleic acid, an unsaturated fatty acid [24]. This result aligns with the microstructural observations presented in Figure 1 and Figure 2. The complex index of the MS group increased significantly after microwave treatment, unlike the MO group. Both groups reached their highest complex index after two minutes of treatment, followed by a decline at three minutes. Microwave irradiation, due to its high penetrative power, causes polar water molecules in the sample to oscillate continuously, generating friction and substantial heat energy [25]. The literature has reported that the single-helical structure of amylose can fully unravel at high temperatures around 160 °C [26], which may facilitate the formation of V-type single-helical complexes induced by fatty acids. Additionally, another study comparing complexation conditions at 121 °C and 95 °C showed that higher temperatures are more favorable for complex formation [27]. In this study, the sample temperature after microwave post-treatment ranged from 110 to 120 °C, with localized hot spots potentially reaching even higher temperatures. The elevated temperature of the aqueous medium likely improved the solvent quality of water for amylose by disrupting its helical memory, thereby increasing its availability for complex formation. However, these high temperatures may have also induced partial depolymerization of starch chains. The rapid energy accumulation could exert stronger disruptive effects on α-1,6-glycosidic bonds due to their large steric hindrance and structural instability [28], thereby promoting the formation of relatively linear starch chains. Although some of the resulting depolymerized molecules may have a degree of polymerization (DP) too low for effective complex formation, and may also retain some branching, the longer depolymerized amylopectin chains may still participate in complexation with fatty acids, provided the chain length is sufficient [29]. These starch–lipid complexes could aggregate into crystalline structures, thereby increasing the overall complexing index [29]. However, prolonged microwave treatment (three minutes) resulted in a significant decrease in the complex index, likely due to the extensive cleavage of amylopectin side chains. These shorter chains may bind iodine more effectively, increasing the absorbance values and consequently reducing the calculated complex index.

### 3.4. Long-Range Ordered Structure by X-Ray Diffraction (XRD) Analysis

**Figure 3:**
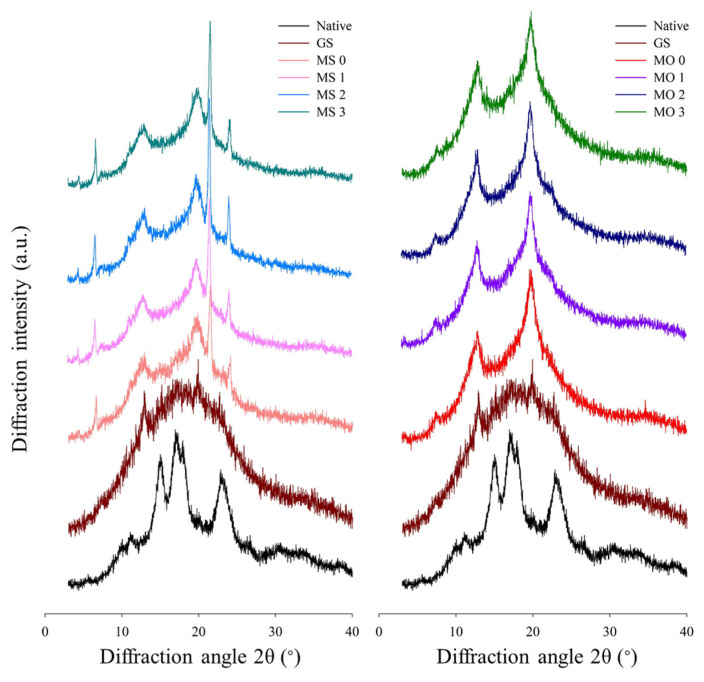
XRD diffraction patterns of native, gelatinized water caltrop starch, and microwave post-treated starch–lipid complexes. Sample codes are the same as described in Table 1.

Figure 3 shows the X-ray diffraction patterns of native, gelatinized water caltrop starch, and microwave post-treated starch–lipid complexes. Native water caltrop starch exhibited diffraction peaks at 2θ of 9.9°, 11°, 15°, 17°, 18°, and 23°, characteristic of an A-type crystalline structure, which is consistent with previous studies [30,31]. After the gelatinization process under sufficient heat and excess water conditions, the semi-crystalline structure of the starch, primarily composed of amylopectin, theoretically should be completely destroyed, leading to the disappearance of distinct diffraction peaks. However, as shown in Figure 3, the gelatinized water caltrop starch sample shows slight but noticeable characteristic diffraction peaks of V-type crystallinity at 2θ of 13° and 19.8°, suggesting the complex formation between amylose and ethanol during the ethanol washing process after gelatinization [32,33]. This result aligns with the microstructural observations presented in Figure 1. With the addition of fatty acids, both the MS and MO groups exhibited characteristic V-type crystallization diffraction peaks at 2θ values of 7.5°, 13°, and 19.8° [13], indicating the successful formation of V-type complexes. Le, et al. [34] reported that the crystalline arrangement of V-type starch–lipid complexes varies depending on the guest molecule, reaction medium, and complexation temperature. Common forms include V6I, V6II, V7, or mixed types composed of any two of these structures. In the present study, the preparation conditions—namely, the use of long-chain fatty acids (carbon number > 10), water as the reaction medium, and a complexation temperature of 90 °C—are comparable to those previously reported. Moreover, the X-ray diffraction pattern of the resulting complexes exhibits characteristic peaks at 7.5°, 13°, and 19.8°, which are consistent with the typical V6I-type structure. Therefore, the starch–lipid complexes formed in this study can be reasonably classified as V6I-type. It should be noted that while ethanol can form V6-type complexes with amylose, its effectiveness depends on both concentration and processing temperature. In this study, ethanol was used to pre-dissolve fatty acids prior to complexation and to wash off unbound fatty acids after the reaction. The total amount of ethanol used for pre-dissolution was substantially lower than the concentration required to induce amylose–ethanol complexation [35]. Moreover, the complexation reaction was conducted at 90 °C, a temperature at which most free ethanol likely evaporated. The greater hydrophobicity of fatty acids compared to ethanol further suggests that complexation was primarily driven by interactions between amylose and fatty acids. Additionally, although a large volume of ethanol (200 mL) was used during the washing step, it was applied at a reduced temperature, further minimizing the potential for ethanol-induced complexation. Therefore, ethanol’s influence on the overall yield of V-type complexes with fatty acids is considered minimal. However, for the MS group, despite ethanol washing to remove free fatty acids, thin diffraction peaks at 2θ of 6.5°, 21.5°, and 24° indicate residual uncomplexed stearic acid. Additionally, as shown in Table 2, the relative crystallinity (RC%) of native water caltrop starch was found to be 44.5%, which is comparable with previous research on water caltrop starch [30,31]. For gelatinized starch, the RC% was found to be 4.5%, possibly due to the complex formed between amylose and ethanol during the process of precipitating the gelatinized starch with 95% ethanol as well as retrogradation during the subsequent drying process. The RC% of the complexes formed between water caltrop starch and stearic acid or oleic acid were 16.8% and 19.4%, respectively. After microwave treatment, the RC% of both the starch–stearic acid (MS) and starch–oleic acid (MO) groups significantly increased. The change in relative crystallinity (RC%) of the water caltrop starch complexes was calculated by subtracting the RC% of the 0-min treatment sample from that of the 3-min treatment sample. The increase in RC% was more pronounced in the MS group, which rose by 10.4% (from 16.8% to 27.2%), compared to a 4.8% increase in the MO group (from 19.5% to 24.3%). This suggests that microwaves have a more pronounced effect on the complex formed with stearic acid. The increase in RC% suggests that microwave exposure promotes a more ordered arrangement of starch–lipid complexes. Microwave irradiation promotes the vibration and friction of polar molecules within the sample, thereby enhancing interactions between amylose and fatty acids [36]. Simultaneously, the vigorous movement of water molecules generates localized hot spots, creating a high-temperature microenvironment that favors starch–lipid complex formation. These hot spots facilitate the conversion of microwave energy into heat, resulting in rapid energy accumulation [28]. Consequently, structurally less stable α-1,6-glycosidic bonds may undergo depolymerization, producing relatively linear starch chains that can further interact with free fatty acids to form complexes. These microwave-induced complexes may then organize into ordered crystalline structures, contributing to the observed increase in relative crystallinity (RC%).

### 3.5. Short-Range Ordered Structure by Fourier-Transform Infrared Spectroscopy (FTIR) Analysis

**Figure 4:**
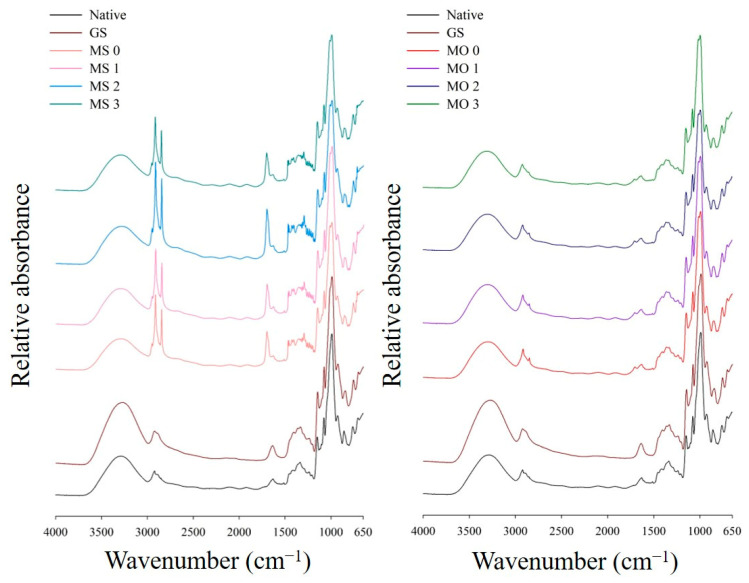
FTIR spectra of native, gelatinized water caltrop starch, and microwave post-treated starch–lipid complexes. Sample codes are the same as described in Table 1.

As shown in Figure 4, all samples, including native and gelatinized water caltrop starch, and the starch–lipid complex groups, exhibited a broad band in the range of 3600 cm^−1^ to 3000 cm^−1^, corresponding to the stretching vibrations of hydroxyl (–OH) groups [35]. In both the MS and MO complex groups, distinct peaks were observed at 2920 cm^−1^, 2850 cm^−1^, and 1700 cm^−1^, attributed to the asymmetric stretching vibration of –CH_2_, the stretching vibration of C–H, and the stretching vibration of C=O, respectively [14]. However, the MS group exhibited stronger peaks corresponding to the carbonyl and alkyl stretching vibrations of fatty acids, possibly due to the presence of residual free stearic acid after ethanol washing, as also indicated by the XRD results shown in Figure 3.

The wavenumbers 1047 cm^−1^, 1022 cm^−1^, and 995 cm^−1^ are commonly used to assess the short-range molecular structure of starch. The peak at 1047 cm^−1^ corresponds to crystalline regions, 1022 cm^−1^ to amorphous regions, and 995 cm^−1^ reflects the presence of hydrogen bonds within the starch structure. As shown in Table 3, gelatinization disrupts the crystalline regions, resulting in a decreased 1047 cm^−1^/1022 cm^−1^ ratio and reduced short-range order. Neither the type of fatty acid nor microwave treatment significantly affected the 1047/1022 cm^−1^ ratio of the starch–lipid complex, likely due to the structural heterogeneity of the complexes. This observation is consistent with the findings of Nie, et al. [37]. However, the addition of stearic and oleic acids to gelatinized starch significantly decreased the 995 cm^−1^/1022 cm^−1^ ratio, which reflects the number of double helices in starch. This confirms that the formation of amylose–lipid complexes results in a reduction in double helices. This observation is consistent with the findings of Zheng, et al. [38].

### 3.6. Thermal Properties by Differential Scanning Calorimetry (DSC) Analysis

**Figure 5:**
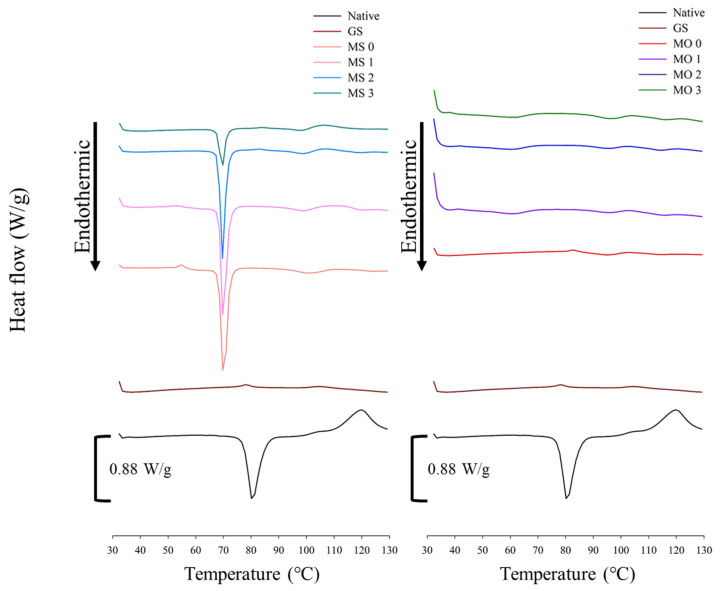
DSC thermograms of native, gelatinized water caltrop starch and microwave post-treated starch–lipid complexes. Sample codes are the same as described in Table 1.

As shown in Figure 5, native starch showed an endothermic peak around 80 °C, which corresponds to the gelatinization transition and is consistent with previous studies of water caltrop starch [30,31]. For gelatinized starch, no detectable endothermic peak was observed. In the MS complex group, the endothermic transition at 69 °C is indicative of the melting of stearic acid. Both the MS and MO complex groups exhibited additional endothermic peaks above the starch gelatinization temperature, attributed to the melting of Type I and Type II starch–lipid complex crystals. Starch–lipid complexes form crystalline structures via stacking, and the specific arrangement of these structures influences their thermal stability. Type I complex crystals typically dissociate at 90–105 °C, while Type II complex crystals are more thermally stable, dissociating at 105–130 °C [20,39,40,41].

Table 4 summarizes the onset (T_o_), peak (T_p_), and final melting temperatures (T_e_), melting temperature difference (T_e_ − T_o_), and enthalpy (ΔH) of the starch–lipid V-type crystalline complexes (peaks observed above the completion temperature of the gelatinization transition (86 °C) of water caltrop starch). The starch–stearic acid complex without microwave post-treatment (MS 0) showed peaks at 100.11 °C and 121.17 °C, while the starch–oleic acid complex without microwave post-treatment (MO 0) exhibited peaks at 94.93 °C and 113.4 °C, indicating that both saturated and unsaturated fatty acids can form Type I and Type II V-type crystals with water caltrop starch. However, the starch–stearic acid complex demonstrated higher melting temperatures and enthalpy (notably for Type I V-type crystals), suggesting greater thermal stability and energy required for melting, consistent with reports that thermal stability decreases with increasing fatty acid unsaturation [39]. Microwave post-treatment significantly altered the thermal properties of starch–lipid complexes. In the MS group, increasing microwave exposure decreased the melting temperatures, melting temperature range, and enthalpy change (ΔH) of Type I crystals. This is likely due to enhanced interactions between free fatty acids and uncomplexed amylose or pseudo-amylose formed by the cleavage of amylopectin side chains [35]. These interactions may lead to the formation of more microwave-induced complexes with lower melting points and greater uniformity, as indicated by the reduced (T_e_ − T_o_) values [42]. Although the melting temperatures of Type II crystals also decreased, their melting ranges and ΔH increased, possibly due to the rearrangement of Type I complexes into less ordered Type II crystals under microwave energy. These newly formed regions may still bind fatty acids but exhibit lower crystallinity. In contrast, the Type I and Type II endothermic peaks of the MO group were not significantly affected by microwave treatment, apart from a slight increase in enthalpy. The lower melting temperature of Type I crystals in the oleic acid complexes may be attributed to the presence of double bonds, which reduce molecular linearity, weaken amylose interactions, and decrease thermal stability [39]. Consequently, microwave treatment induced fewer new complexes in the MO samples, thereby limiting its impact on their thermal properties.

### 3.7. Mechanism of Microwave Post-Treatment on the Construction of Starch–Lipid Complexes

**Figure 6:**
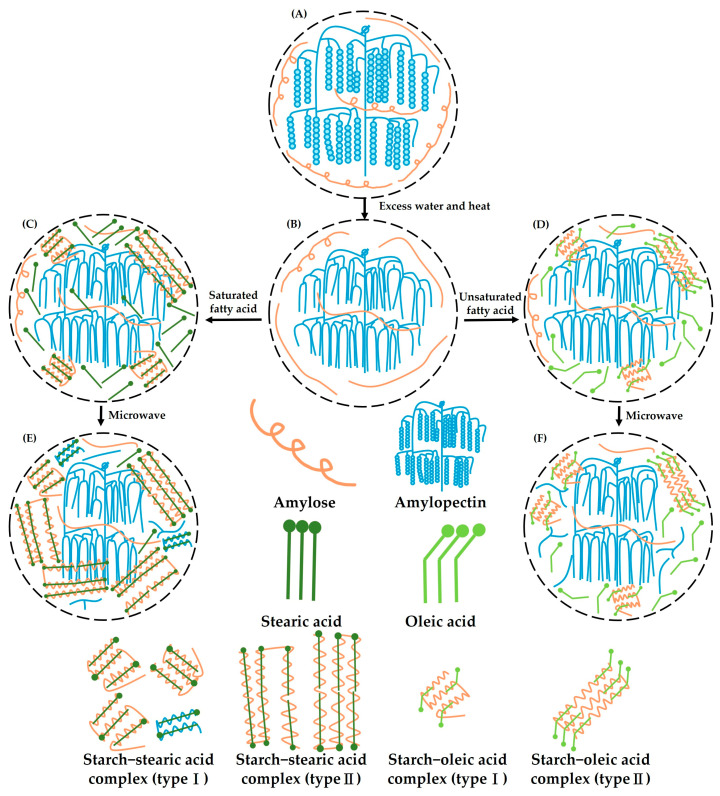
Mechanism of microwave post-treatment on the construction of starch–stearic acid complex and starch–oleic acid complex. (**A**) Native water caltrop starch, (**B**) gelatinized water caltrop starch, (**C**,**D**) starch–lipid complex without microwave post-treatment, (**E**,**F**) starch–lipid complex with microwave post-treatment.

Figure 6 illustrates the proposed mechanism of microwave post-treatment on the formation of starch–stearic acid and starch–oleic acid complexes. The crystalline regions of water caltrop starch are composed of amylopectin side chains arranged in double-helix structures, whereas amylose is primarily located in the amorphous regions, existing mainly in a single-helix conformation, with a smaller fraction in a flexible linear form (Figure 6A). When sufficient moisture is present and the temperature exceeds the gelatinization threshold, the crystalline regions begin to be disrupted, leading to the unraveling of the double-helix structures. Simultaneously, much of the amylose in the single-helix form also tends to unfold (Figure 6B). This structural unfolding allows fatty acids to induce the formation of amylose single helices, which then organize into ordered crystalline complexes. Both stearic and oleic acids can form complexes with water caltrop starch that contain Type I and Type II crystalline structures (Figure 6C,D).

Localized hot spots generated by microwave treatment further improved the solvent quality of water for amylose by disrupting its helical memory, and promoted the depolymerization of amylopectin, thereby increasing its availability for complex formation with stearic acid. This process leads to the formation of more uniform Type I crystals with lower melting temperatures. Simultaneously, portions of the original Type I complexes that were not initially bound to fatty acids may reorganize with free stearic acid into less uniform Type II crystals with reduced melting temperatures (Figure 6E). In contrast, microwave treatment had a relatively limited effect on starch–oleic acid complexes. Although the high temperatures generated during microwave heating may cause the single-helical conformation of amylose to unravel and enable subsequent interaction with oleic acid, only a small number of new type I complexes were formed. This limited formation is likely due to the molecular kink in oleic acid, caused by its double bond, which reduces linearity and hinders tight packing (Figure 6F). After three minutes of microwave treatment, further cleavage of amylopectin generates more short-chain starch molecules, which, if not bound to fatty acids, remain uncomplexed in both types of starch–lipid systems.

## 4. Conclusions

The starch–stearic acid complex exhibits more pronounced sheet-like structures, birefringent spots, and a higher complex index (78.8%) compared to the starch–oleic acid complex (77.3%). X-ray diffraction analysis confirmed that native water caltrop starch possesses an A-type crystalline structure, which transforms into a V-type pattern following gelatinization and fatty acid addition, indicating the formation of starch–lipid complexes. Microwave treatment had a more pronounced effect on the long-range crystalline order, as evidenced by increased relative crystallinity, than on the short-range molecular structure, as assessed by FTIR. This effect was more pronounced in the stearic acid complex, likely due to the saturated nature of stearic acid facilitating tighter molecular packing and more organized structures. Both fatty acids formed Type I and Type II crystalline complexes with starch; however, stearic acid complexes demonstrated higher thermal stability, attributed to their larger and more ordered crystalline domains. Short-term microwave treatment further enhanced this organization, possibly by promoting interactions between stearic acid and short-chain amylose generated during heating. In contrast, the oleic acid complex was less responsive to microwave treatment, with its molecular kink caused by the double bond reducing linearity and hindering tight packing. Consequently, it exhibited lower thermal stability, weaker crystalline organization, and fewer sheet-like structures observed via SEM. While this study advances understanding of how microwave treatment and fatty acid type influence starch–lipid complex structure, further research is necessary to evaluate their digestibility and rheological properties to support potential applications in food processing.

## Figures and Tables

**Table 1:** Complex index of native, gelatinized water caltrop starch, and microwave post-treated starch–lipid complexes.

Sample ^1^	Complex Index (%) ^2^
Native	- ^3^
GS	- ^3^
MS 0	78.8 ± 0.1 ^c^
MS 1	79.5 ± 0.1 ^b^
MS 2	80.1 ± 0.2 ^a^
MS 3	79.8 ± 0.2 ^ab^
MO 0	77.3 ± 0.1 ^d^
MO 1	77.3 ± 0.3 ^d^
MO 2	77.8 ± 0.2 ^d^
MO 3	75.7 ± 0.1 ^e^

^1^ “Native” denotes native water caltrop starch, “GS” denotes gelatinized water caltrop starch, “MS” denotes water caltrop starch–stearic acid complex, and “MO” denotes water caltrop starch–oleic acid complex, respectively. The number after MS and MO denotes the duration of microwave post-treatment in minutes. ^2^ Each data is expressed as the mean ± standard deviation. Means in the same column with different superscript letters are significantly different (*p* < 0.05). ^3^ Not applicable.

**Table 2:** Relative crystallinity and crystalline types of native, gelatinized water caltrop starch, and microwave post-treated starch–lipid complexes.

Sample ^1^	Relative Crystallinity (%) ^2^	Crystalline Structure
Native	44.5 ± 0.4 ^a^	A-type
GS	4.5 ± 0.6 ^f^	V-type
MS 0	16.8 ± 0.4 ^e^	V-type
MS 1	19.4 ± 0.8 ^d^	V-type
MS 2	20.2 ± 0.5 ^d^	V-type
MS 3	27.2 ± 0.7 ^b^	V-type
MO 0	19.5 ± 0.3 ^d^	V-type
MO 1	23.3 ± 0.3 ^c^	V-type
MO 2	23.4 ± 0.3 ^c^	V-type
MO 3	24.3 ± 0.3 ^c^	V-type

^1^ Sample codes are the same as described in Table 1. ^2^ Each data is expressed as the mean ± standard deviation. Means in the same column with different superscript letters are significantly different (*p* < 0.05).

**Table 3:** FTIR ratio of native, gelatinized water caltrop starch and microwave post-treated starch–lipid complexes ^1−2^.

Sample ^1^	1047 cm^−1^/1022 cm^−1^	995 cm^−1^/1022 cm^−1^
Native	0.711 ± 0.002 ^a^	1.278 ± 0.000 ^a^
GS	0.685 ± 0.002 ^b^	1.143 ± 0.002 ^b^
MS 0	0.656 ± 0.001 ^c^	1.095 ± 0.003 ^cd^
MS 1	0.668 ± 0.014 ^bc^	1.100 ± 0.008 ^c^
MS 2	0.674 ± 0.028 ^bc^	1.082 ± 0.001 ^d^
MS 3	0.676 ± 0.006 ^bc^	1.102 ± 0.001 ^c^
MO 0	0.651 ± 0.008 ^c^	1.108 ± 0.007 ^c^
MO 1	0.656 ± 0.003 ^c^	1.101 ± 0.006 ^c^
MO 2	0.655 ± 0.007 ^c^	1.098 ± 0.002 ^c^
MO 3	0.664 ± 0.011 ^bc^	1.092 ± 0.014 ^cd^

^1^ Sample codes are the same as described in Table 1. ^2^ Each data is expressed as the mean ± standard deviation. Means in the same column with different superscript letters are significantly different (*p* < 0.05).

**Table 4:** Thermal parameters of microwave post-treated starch–lipid complexes from the endothermic peaks above 86 °C in DSC.

			Type Ⅰ					Type Ⅱ		
Sample ^1^	T_o_^2,3^ (℃)	T_p_^2,3^ (℃)	T_e_ ^2,3^ (℃)	T_e_ − T_o_ ^2,3^ (℃)	ΔH ^2,3^ (J/g)	T_o_^2,3^ (℃)	T_p_^2,3^ (℃)	T_e_ ^2,3^ (℃)	T_e_ − T_o_ ^2,3^ (℃)	ΔH ^2,3^ (J/g)
Native	- ^4^	- ^4^	- ^4^	- ^4^	- ^4^	- ^4^	- ^4^	- ^4^	- ^4^	- ^4^
GS	- ^4^	- ^4^	- ^4^	- ^4^	- ^4^	- ^4^	- ^4^	- ^4^	- ^4^	- ^4^
MS 0	92.41 ± 0.11 ^a^	100.11 ± 0.41 ^a^	108.42 ± 0.06 ^a^	16.01 ± 0.04 ^a^	7.81 ± 0.07 ^a^	117.42 ± 2.1 ^a^	121.17 ± 1.7 ^a^	124.97 ± 0.96 ^a^	7.55 ± 1.14 ^b^	0.39 ± 0.06 ^b^
MS 1	92.21 ± 0.00 ^ab^	98.49 ± 0.1 ^b^	104.76 ± 0.65 ^b^	12.55 ± 0.65 ^b^	4.15 ± 0.3 ^b^	115.47 ± 0.72 ^a^	119.21 ± 0.45 ^ab^	122.77 ± 0.16 ^a^	7.3 ± 0.88 ^b^	0.95 ± 0.04 ^a^
MS 2	91.96 ± 0.27 ^b^	98.82 ± 0.6 ^b^	105.43 ± 1.89 ^ab^	13.46 ± 1.61 ^ab^	4.81 ± 0.82 ^b^	114.21 ± 0.26 ^a^	118.79 ± 2.24 ^ab^	122.64 ± 0.87 ^a^	8.43 ± 0.6 ^b^	1.03 ± 0.21 ^a^
MS 3	90.04 ± 0.04 ^c^	97.81 ± 0.12 ^b^	103.53 ± 0.97 ^b^	12.57 ± 1.02 ^b^	4.45 ± 0.63 ^b^	108.68 ± 0.49 ^b^	115.95 ± 1.29 ^b^	122.61 ± 1.83 ^a^	13.93 ± 1.33 ^a^	1.00 ± 0.00 ^a^
MO 0	87.61 ± 0.24 ^A^	94.93 ± 0.74 ^A^	100.56 ± 0.32 ^B^	12.94 ± 0.07 ^A^	1.99 ± 0.14 ^A^	108.78 ± 0.42 ^A^	113.4 ± 0.27 ^B^	117.59 ± 0.38 ^A^	8.81 ± 0.8 ^B^	0.51 ± 0.04 ^D^
MO 1	87.57 ± 0.76 ^A^	95.38 ± 0.12 ^A^	101.27 ± 0.12 ^AB^	13.7 ± 0.89 ^A^	1.86 ± 0.24 ^A^	107.85 ± 0.98 ^A^	114.67 ± 0.82 ^AB^	119.35 ± 0.93 ^A^	11.49 ± 0.04 ^A^	1.1 ± 0.49 ^B^
MO 2	87.48 ± 0.76 ^A^	95.18 ± 0.34 ^A^	100.8 ± 0.38 ^B^	13.32 ± 0.37 ^A^	1.78 ± 0.06 ^A^	108.79 ± 1.55 ^A^	114.23 ± 0.7 ^AB^	118.8 ± 1.03 ^A^	10.01 ± 0.51 ^B^	1.24 ± 0.02 ^A^
MO 3	88.25 ± 0.11 ^A^	95.44 ± 0.45 ^A^	101.79 ± 0.12 ^A^	13.54 ± 0.00 ^A^	2.25 ± 0.14 ^A^	109.95 ± 0.04 ^A^	115.24 ± 0.22 ^A^	119.3 ± 0.23 ^A^	9.31 ± 0.18 ^B^	0.81 ± 0.02 ^C^

^1^ Sample codes are the same as described in Table 1. ^2^ T_o_ indicates onset temperature, T_p_ indicates peak temperature, T_e_ indicates endset temperature, T_e_ − T_o_ indicates the temperature difference between T_e_ and T_o_ and ∆H indicates the transition enthalpy. ^3^ Each data is expressed as the mean ± standard deviation. Means in the same column with different superscript letters are significantly different (*p* < 0.05). Uppercase and lowercase letters indicate the statistical differences in the results of starch–oleic acid complex and starch–stearic acid complex, respectively.^4^ Not detected.

## Data Availability

The data presented in this study are available on request from the corresponding author. The data are not publicly available due to ethical restriction and the intellectual property issue.
